# Role of Woody Biomass Ash Material in Immobilization of Cadmium, Lead and Zinc in Soil

**DOI:** 10.3390/ma17102206

**Published:** 2024-05-08

**Authors:** Elżbieta Rolka, Mirosław Wyszkowski, Andrzej Cezary Żołnowski, Anna Skorwider-Namiotko

**Affiliations:** Department of Agricultural and Environmental Chemistry, Faculty of Agriculture and Forestry, University of Warmia and Mazury in Olsztyn, Łódzki 4 Sq., 10-727 Olsztyn, Poland; elzbieta.rolka@uwm.edu.pl (E.R.); andrzej.zolnowski@uwm.edu.pl (A.C.Ż.); anna.namiotko@uwm.edu.pl (A.S.-N.)

**Keywords:** ash from wood biomass, soil contamination, immobilization, cadmium, lead, zinc

## Abstract

Nowadays, we have observed the dynamic development of bio-heating plants that use wood biomass for heating or energy purposes. The result of this process is a reduction in carbon dioxide emissions as well as in the production of biomass ash (BA). Despite the waste nature of BA, it should be carefully analyzed and assessed for various applications, including environmental ones. Due to the features attributed to BA, including its alkaline reaction, the high capacity of its sorption complex, relatively low salinity, and significant content of macro- and microelements, a hypothesis was put forward in this work undertaken about the positive role of BA as an immobilizing factor for Cd-, Pb-, and Zn-contaminated soils. This research was based on a pot experiment in which four series were considered: (1) BA; (2) BA + Cd; (3) BA + Pb; and (4) BA + Zn. BA was used at doses of 30, 60, and 90 mg pot^−1^, and metals at doses of 2 mg Cd, 100 mg Pb, and 300 mg Zn kg^−1^ of soil. The test plant was corn grown for green mass. The study took into account the influence of BA on the content of the total forms of heavy metals (Me_tot_) and their available forms (Me_av_). In the soil without the addition of metals, a significant increase in the content of Cd_tot_ and Cd_av_, and a decrease in the content of Zn_tot_ were observed due to the application of BA. The addition of metals against the background of the BA used resulted in a significant increase in Cd_tot_, Pb_tot_, and Zn_tot_, as well as an increase in the available forms of Pb_av_ but a decrease in Zn_av_. However, there was no significant increase in the Cd_av_ content. The obtained results may indicate the potentially immobilizing role of BA only in the case of zinc. They may constitute the basis for further, more detailed research aimed at determining the role of BA in the immobilization of various metals in soil.

## 1. Introduction

In the search for effective materials capable of absorbing heavy metals from the environment, including the soil environment, special attention should be paid to waste materials or byproducts of industrial processes. Such processes among others include biomass incineration for heating or energy purposes. The use of biomass in energy production is aimed at reducing the emission of carbon oxides, sulfur dioxide, nitrogen oxides, and other toxic substances into the environment, compared to classic solid and liquid fossil energy sources [1,2], which is one of the basic assumptions of the “Polish Energy Policy until 2030” program [2]. It is estimated that by 2050, 33% to 50% of global energy resources could be covered by biomass burning [3]. The biomass combustion process on a global scale reduces exhaust gas emissions into the environment; however, at the same time it generates a byproduct in the form of biomass ash (BA) [3]. Among the types of biomasses, wood biomass in the form of wood chips is most often used for heating purposes. From the emission point of view, the combustion of wood chips is not included in the carbon dioxide (CO_2_) emission limits. The use of biomass in the energy sector reduces the accumulative carbon footprint [4]. However, the rational use of produced BA turns out to be problematic. This is because biomass ash (BA) has a variable composition, which is influenced not only by the type of biomass burned [1,2,3,5,6] and its storage time [6], but also by the temperature during the combustion process [2,3] and the possible treatment of the biomass before combustion [1]. According to Zając et al. [5], an increase in the temperature of the wood biomass combustion process reduces the contents of Zn, Pb, Cd, and Cu in BA and increases the content of Cr, Ni, and Fe. Nzihou and Stanmore [7] claim that all metals except Hg are retained in BA. According to the authors, at temperatures below 1000 °C, metals are evenly distributed in each BA particle. However, at temperatures higher than 1000 °C, the external shell of dust molecules is decomposed and smaller particles containing higher concentrations of trace elements are formed. Higher metal concentrations in the finer BA particles result from the devolatilization of metals and their re-condensation. According to Munawar et al. [4], BA storage and transportation also promote the transformation of compounds contained in BA, which react with each other through oxidation and dehydration, creating new, complex compounds.

If BA comes from a reliable source that guarantees a pure and homogeneous raw material, then it contains specific, stable amounts of minerals and trace elements [4]. Due to the abundance of BA in nutrients, including macroelements (Ca, K, Mg, P, Na) and microelements (Zn, Fe, Mn, Cu, B), the application of this material to the soil should be considered first, as it has been proven to have a positive effect on soil properties [4,8]. BA, characterized by a negligible level of xenobiotics, can be used as a substitute for lime in agriculture or for the reclamation of degraded land to improve soil properties. Some authors propose the use of BA as a binding material in the process of fertilizer production from other waste, including sewage sludge [3]. The environmentally friendly management of BA would classify them as raw materials rather than waste and would also promote sustainable development [4] in line with the assumptions of the circular economy. However, the long-term application of BA to soil may become a source of pollution or even contamination of soils due to the presence of heavy metals and other inorganic compounds [1,5] as well as polycyclic aromatic hydrocarbons (PAHs) [3]. Moreover, the process of the compounds leaching from BA after their application to the soil still needs to be clarified [3].

This present work draws attention to the aspect related to the possibility of using BA in soils with increased Cd, Pb, and Zn content. The focus was on the above-mentioned elements because, for example, Cd is characterized by a significant degree of dispersion in bedrocks, high mobility in the environment, and easy movement in the food chain [9,10]. The contamination of soil resources with Cd is constantly increasing, and the contribution of anthropogenic activities in this aspect is very high [10]. In turn, Pb, although considered a less mobile element, is also closely related to the mineralogical and granulometric composition of soils and the parent rock. Pb migrates equally easily in the food chain, especially in soil-contaminated conditions, and, like Cd, it is a toxic element to living organisms. However, Zn is characterized by high solubility in soil solution. Zn has a double meaning: on the one hand, it is a very important microelement in plant nutrition and an essential supplement to the human diet. In plant cultivation, both deficiency and excess of this element may be harmful [9]. The mentioned metals are constantly emitted into the environment as a result of anthropogenic activities such as the rapidly developing metallurgical industry, improper waste disposal, the use of fertilizers and plant protection products, and the use of municipal sewage sludge in agriculture. Their harmfulness and health effects have been widely described in the literature [11]. Moreover, a common feature of these elements is that the acidic reaction of soils favors their mobility in the environment [9]. This knowledge enables the search for appropriate methods of their immobilization in the soil, based on the use of sorbent materials with similar properties. Ultimately, this facilitates the selection of immobilizing additives used for soils contaminated with various metals without causing contradictory effects on their mobility, bioavailability, and, importantly, toxicity [12]. There is relatively little information in the literature on the immobilization role of BA that these materials can play in relation to toxic substances contained in the soil. However, the potential of BA in improving soil properties associated with an increase in pH is quite well known [4,8], as it is able to increase the sorption complex [8] and increase retention in relation to nutrients [8,13,14] and water [4]. The above information provides the basis for indicating the possibility of immobilizing heavy metals in soils as a result of the soil application of BA. However, it should be admitted that most of the research results published so far have not taken into account the properties of BA in relation to the immobilization of heavy metals in the soil. Rather, BA is considered to be a significant source of heavy metals which it can release into the soil during its application [1]. Additionally, the storage of BA can in turn cause the migration of metals and other pollutants not only to soils but also to waters [6]. Due to the few mentions in the literature about the potential role of BA in Cd immobilization [8], the purpose of the research undertaken was to verify these few reports.

In line with these considerations, a research hypothesis was formulated in this study stating that BA may be a factor in immobilizing Cd, Pb, and Zn in soil with increased content of these metals, compared to the null hypothesis stating that there is no effect of BA on the content of the total and available forms of heavy metals in the soil. The primary aim of this work was to demonstrate the possibility of the immobilization of Cd, Pb, and Zn in soil as a result of BA application. The specific objective was to verify whether the adopted doses of BA in conditions of increased metal content would prove effective in immobilizing these elements.

## 2. Materials and Methods

### 2.1. Experiment Description

This research was based on a pot experiment, which was carried out in a vegetation hall located on the campus of the University of Warmia and Mazury in Olsztyn (Poland). The experiment included four series: (1) BA, (2) BA + Cd, (3) BA + Pb, and (4) BA + Zn. In all series, ash from wood biomass combustion (BA) was used as a potential Cd-, Pb-, and Zn-immobilizing additive at doses of 30, 60, and 90 g pot^−1^. The simulated soil pollution with metals was 2 mg Cd, 100 mg Pb, and 300 mg Zn kg^−1^ soil. Each series included a control subject without the addition of BA. The mass of soil used in the experiment was 9 kg pot^−1^.

Metals were introduced in the form of aqueous solutions of cadmium acetate (Cd(CH_3_COO)_2_ 2H_2_O) (POCh Gliwice, Poland), lead acetate (Pb(CH_3_COO)_2_ 3H_2_O) (POCh Gliwice, Poland), and zinc acetate (Zn(CH_3_COO)_2_ 2H_2_O) (Chempur, Piekary Śląskie, Poland). Mineral fertilization was applied to each pot, regardless of the series: 0.111 g N, 0.113 g K, and 0.067 g P kg^−1^ soil. N was applied in the urea form (CH_4_N_2_O), P in the form of ammonium phosphate (NH_4_)_2_HPO_4_), and K as a potassium sulfate (K_2_SO_4_).

The metal levels used in the experiment (Cd, Pb, and Zn), according to the Regulation of the Minister of the Environment of 1 September 2016 [15], were the maximum allowable contents of these elements in agricultural soils in Poland. According to the mentioned act, these are the maximum permissible concentrations, which according to the Institute of Soil Cultivation and Fertilization in Puławy [16] are referred to as “increased contents”.

In total, the experiment included 16 treatments. Each treatment was considered in three replications. In the experiment, corn (*Zea mays* L.) was grown for green mass for a period of 51 days (from June 14 to August 4). After harvesting the plants, soil samples were taken from each pot, then dried in the open air and sieved through a sieve with a mesh diameter of 2 mm. Samples prepared in this way were stored in paper packages until chemical analyses were performed.

### 2.2. Soil Properties

The soil used in the research had a low pH = 4.2 (measured in 1 mol KCl) and the specific electric conductivity (EC) of 0.019 dS m^−1^. According to its mineralogical composition, the soil was classified as proper brown soil [17], which is typical for most areas of the Warmian–Masurian province (Poland). The soil was characterized by a poor sorption complex (SC). The sum of base cations (SBC), hydrolytic acidity (HAC), and cation exchange capacity (CEC) were 34.5, 22.0, and 56.50 mmol(+) kg^−1^ of soil, respectively. The base saturation (BS) was only 38.73%. The total carbon content (C_tot_) and total nitrogen content (N_tot_) were 4.377 g and 0.562 g kg^−1^, respectively, and the C/N ratio was 7.79. The content of available forms of phosphorus (P_av_), potassium (K_av_), and magnesium (Mg_av_) were 79.03; 123.5, and 21.13 mg kg^−1^ of soil, respectively. The content of the total forms of heavy metals Cd_tot_, Pb_tot_, and Zn_tot_ were 0.687; 13.22, and 18.57 mg kg^−1^ of the soil, and the available forms of Cd_av_, Pb_av_, and Zn_av_ were 0.130; 6.276, and 9.902 mg kg^−1^ of the soil, respectively.

### 2.3. Biomass Ash (BA) Properties

**Figure 1:**
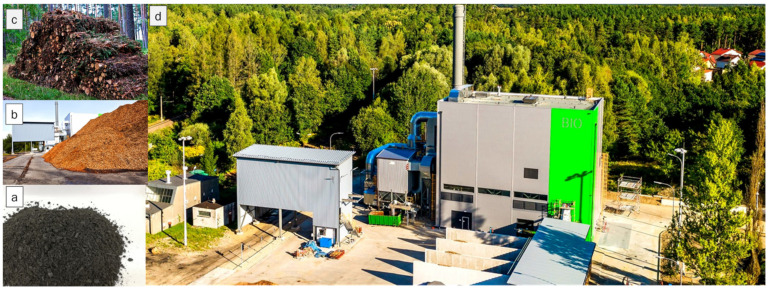
Kortowo-Bio Heating Plant of Municipal Heating Energy Company in Olsztyn (MPEC Olsztyn). (**a**) Biomass ash (BA), (**b**) wood chips, (**c**) forest waste, and (**d**) view of the Kortowo-Bio Heating Plant [18].

The biomass ash (BA) used in the experiment (Figure 1a) originated from Municipal Heating Energy Company in Olsztyn (MPEC Olsztyn, Olsztyn, Poland) and was a by-product of the combustion process of wood chips (Figure 1b), which in Poland are obtained mainly from the processing of coniferous trees, among others that are dominated by species of the genus *Pinus* L. (Figure 1c). These wood chips are burned in one of the boilers located at MPEC Olsztyn—the Kortowo-Bio heating plant (Figure 1d). During the heating season, the Kortowo-Bio Heating Plant burns approximately 50,000 Mg wood chips [18].

Biomass ash (BA), according to the legal acts [19] currently applicable in Poland, is considered to be waste and is classified under the code 10 01 03 [19]. 

According to the performed analyses, BA was classified as an alkaline material (pH_KCl_ = 10.31). The high BA reaction resulted from the level of saturation of the sorption complex (SC) with basic cations (BS), which was 82.38%. The individual SC components were: SBC—3501; HAC—750.0, and CEC—4257 mmol(+) kg^−1^ BA. Moreover, quite high C_tot_ content was recorded as C_tot_—208.0 g kg^−1^. The N_tot_ content was 4.29 g kg^−1^, the C/N ratio—48.48, and EC—4.51 dS m^−1^. The dry matter content (DM) in BA was 80.4%. Among the determined total forms of macronutrients in BA content, K_tot_ (17.18 g kg^−1^) and Ca_tot_ (43.97 g kg^−1^) dominated. Significantly lower levels were found for P_tot_ (6.36 g kg^−1^) and Mg_tot_ (6.20 g kg^−1^), and the lowest content was 1.36 g kg^−1^, which was recorded for Na_tot_. The largest amount of available forms of macronutrient contents in BA was K_av_, followed by P_av_, and the smallest amount was Mg_av_, the contents of which were 410.0, 110.0, and 57.00 mg kg^−1^ of BA, respectively. The contents of Cd_tot_, Pb_tot_, and Zn_tot_ (the elements considered in this work) were 1.50; 74.53, and 448.5 mg kg^−1^ of BA, respectively. In turn, the content of the total forms of other heavy metals in BA: Fe, Mn, Cu, Ni, Co, and Cr, were, respectively, 5684.3, 2377.3, 26.54, 49.95, 4.808, and 30.41 mg kg^−1^ of BA d.m.

### 2.4. List of Chemical Analyses

#### 2.4.1. Biomass Ash (BA)

The ash samples were placed on metal trays and dried in the open air until a constant mass was obtained. Then, in order to obtain a homogeneous structure, the material was crushed in a mortar. The analyses performed included the following: pH, EC, SBC, and HAC; the content of DM; C_tot_; the total forms of macronutrients (N_tot_, P_tot_, K_tot_, Mg_tot_, Ca_tot_ and Na_tot_); and the available forms of P_av_, K_av_, and Mg_av,_ as well as close to the total content of trace elements (Cd_tot_, Pb_tot_, Zn_tot_, Fe_tot_, Mn_tot_, Cu_tot_, Ni_tot_, Cr_tot_ and Co_tot_). 

#### 2.4.2. Soil

The soil was collected before setting up the experiment (initial soil for testing) and separately from each pot after the experiment. All samples were dried in the open air, sieved through a sieve with a mesh diameter of 2 mm, and additionally ground in a mortar. Chemical analyses of the initial soil also included pH; EC; sorption properties (SBC, HAC); the content of Ctot; available forms of P_av_, K_av_, and Mg_av_; as well as the contents of trace elements (Cd_tot_, Pb_tot_, Zn_tot_) and their available forms.

### 2.5. Methodology of Chemical Analyses

**Figure 2:**
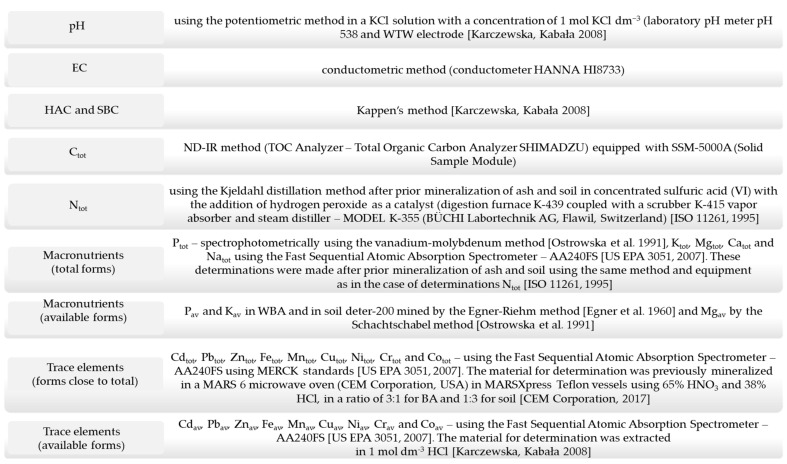
Chemical analyses methods [20,21,22,23,24,25].

Chemical analyses were performed using the following methods (Figure 2).

The results were validated on the basis of the reference material CRM0120-50G (TraceMetals/Sandy Loam 2, SIGMA-ALDRICH Chemie GmbH, Schnelldorf, Germany) and CRM012-100G (TraceMetals/Fly Ash 2, SIGMA-ALDRICH Chemie GmbH, Schnelldorf, Germany). Moreover, the content of heavy metals (total and available forms) was determined using Fluka standard materials (Charlotte, NC, USA, Cd 51994, Co 05202, Cr 02733, Cu 38996, Fe 16596, Mn 63534, Ni 42242, Pb 16595, and Zn 188227).

Based on the determinations of hydrolytic acidity (HAC) and the sum of the bases (SBC) of the analyzed ash and soil samples, the capacity of the sorption complex (CEC) and the degree of saturation of these materials with basic cations (BS) were calculated. However, based on the determination of the content of total forms and available trace elements, the share of available forms in the total content was calculated [26].

### 2.6. Statistical Analysis

The statistical analysis included the LSD test (two-way ANOVA), standard deviation (SD), and Pearson’s simple correlation coefficient (*r*). The LSD test allowed us to assess the impacts of individual BA doses on the possible immobilization of Cd, Pb, and Zn in soil with increased levels of heavy metals. In the LSD analysis, significance and homogeneous groups were determined at the significance level of *p* < 0.05 using the Duncan test. The simple Pearson coefficient (*r*) was used to determine the relationship between the tested features. The estimated coefficient allowed us to determine the direction of the influence of increasing ash doses on the tested features. The significance of the obtained values of the correlation coefficient (*r*) was determined based on statistical tables [27]. Microsoft Excel^®^ for Microsoft 365 MSO, v. 2206 (Microsoft, Redmond, WA, USA) was used for statistical calculations [28] and Statistica v. 13.3 PL (TIBCO Software Inc., Palo Alto, CA, USA) [29]. 

## 3. Results

### 3.1. Contents of the Total Forms of Heavy Metals

The mean Cd_tot_ content in the soil from the series, depending on the series, ranged from 0.588 mg (BA) to 1.910 mg kg^−1^ (BA + Cd) (Table 1). 

The selected series in the experiment indicated that the soil application of BA increased the Cd_tot_ content in the soil. However, a linear and highly significant increase in Cd_tot_ content (r = 0.887 **) was observed only in series 1 (with BA alone), in which the highest BA dose (90 g pot^−1^) had a statistically significant impact on the Cd_tot_ content. Simulated soil contamination in series 2 (BA + Cd), 3 (BA + Pb) and 4 (BA + Zn) additionally increased the Cd_tot_ contents in the soil. However, by far the highest Cd_tot_ content in the soil was recorded in series 2, in which a significant increase in Cd_tot_ content was observed in the treatment with the second dose of BA (60 g pot^−1^).

The mean Pb_tot_ content in the series ranged from 12.88 mg in series 1 (BA) to 81.83 mg kg^−1^ of soil in series 3 (BA + Pb) simulated with Pb pollution (Table 2). In series 1 (with only BA), the BA doses used resulted in a slight increase in the Pb_tot_ content. A significant increase in Pb_tot_ in relation to BA doses was noted only in series 3 (BA + Pb), but only up to the second BA dose (60 g pot^−1^). The highest Pb_tot_ content (94.33 mg kg^−1^ soil) in this series was recorded after the application of the first dose of BA. However, at the highest BA dose, the Pb_tot_ content in the soil was lower than the content in the other objects in this series.

Similar to the soil contents of Cd_tot_ and Pb_tot_, the content of Zn_tot_ was also closely correlated with the experimental series (Table 3).

A similar Zn_tot_ content was recorded in the first three series (BA; BA + Cd; BA + Pb), with a mean ranging from 18.42 to 19.50 mg kg^−1^ of soil. However, in the series with soil simulated as being polluted with Zn (BA + Zn), the Zn_tot_ content in the soil was 10 times higher than the content in the other series and amounted to 195.0 mg kg^−1^ of soil. In turn, the application of BA without the use of a polluting substance resulted in a decrease in the Zn_tot_ content, which can be observed in series 1 (BA). However, in the remaining series, against the background of increasing BA doses, an increase in Zn_tot_ content was noted, as indicated by the correlation coefficient (*r*). In these series, the highest Zn_tot_ content was recorded at the highest BA dose.

### 3.2. Contents of the Available Forms of Heavy Metals

The Cd_av_ content in the soil was strongly correlated with the experimental series (Table 4). The highest content was recorded in series 2 with cadmium acetate (mean 1.538 mg kg^−1^), and in the remaining series, the content was seven to 11 times lower (0.126–0.146 mg Cd_av_ kg^−1^ of soil). The lowest Cd_av_ content was recorded in series 1 with BA alone. The obtained value of the correlation coefficient (*r*) indicated an increase in the Cd_av_ content in the soil as a result of BA application in series 1 (BA) and in series 3 (BA + Pb). However, these differences did not show statistical significance in the LSD test.

The mean content of the available forms of Pb_av_ was at a similar level in series 1 (BA), 2 (BA + Cd), and 4 (BA + Zn), from 6.190 mg to 6.899 mg kg^−1^ (Table 5). A significantly higher mean Pb_av_ content (58.06 mg kg^−1^ soil) was recorded in series 3 with lead acetate. The application of BA-only to the soil increased the Pb_av_ content, but this increase was not statistically significant. Only in the series with lead acetate was a significantly higher Pb_av_ content found in the treatments with BA rather than in the control. The difference in the content between the control and the treatments with BA ranged from 29% to 36%.

Slightly different relationships were observed in the Zn_av_ content in the soil (Table 6). Among the series considered, a significantly higher mean Zn_av_ content was recorded in series 1 (with only BA) and in series 4 (BA + Zn). This content was as follows: 10.70 and 26.95 mg Zn_av_ kg^−1^ soil, respectively. In the remaining series, the Zn_av_ content was much lower; in series 2 (BA + Cd) it was 9.223, and in series 3 (BA + Pb) 8.759 mg kg^−1^ of soil. In the objects with the addition of BA, a higher Zn_av_ content was usually recorded than in the control objects of individual series. These were statistically significant differences. The exception was series 4 (BA + Zn), in which the content of available Zn in the treatments with BA was significantly lower than in the control treatment (without BA).

### 3.3. Correlations—Total Forms vs. Available Forms of Heavy Metals

Pearson’s simple coefficient (*r*) indicated a positive and highly significant correlation between the available forms and the total forms of the considered metal content in the soil (Table 7). The Cd_av_ content was highly significantly correlated to the Cd_tot_ content (*r* = 0.988 **), the Pb_av_ content to Pb_tot_ (*r* = 0.986 **), and the Zn_av_ content to Zn_tot_ (*r* = 0.982 **). In the remaining relations, significant relationships were observed but were of a different negative nature. However, no significant correlation was found in the Cd_tot_ and Zn_tot_ systems.

### 3.4. The Share of the Content of Available Forms of Heavy Metals in Their Total Forms

**Figure 3:**
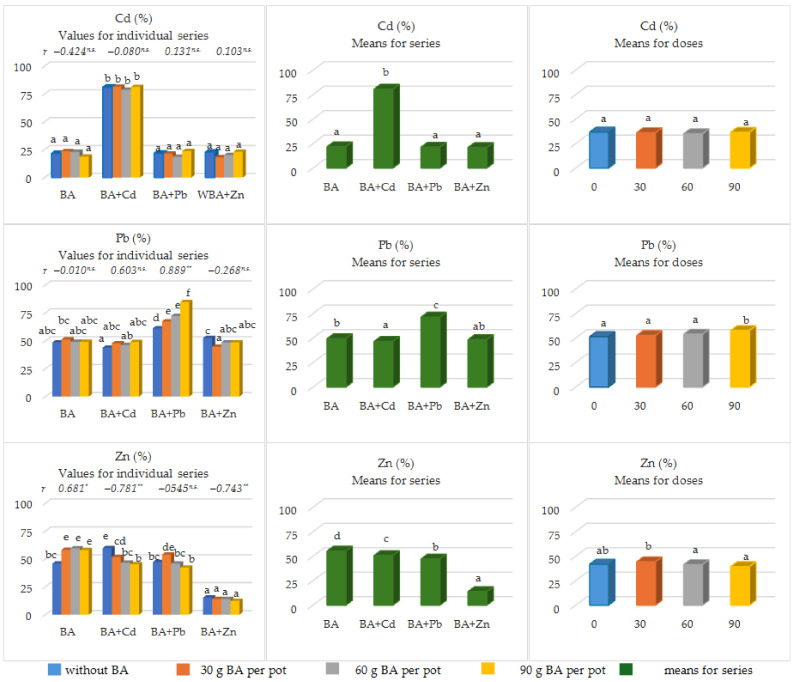
The share of the available forms of heavy metals (Cd, Pb, Zn) in the total pool of the analyzed soils. As part of the LSD*_p_*_ ≤ 0.05_ analysis, different letters (a, b, c, d, abc, etc.) next to the given values indicate the significant impact of individual BA doses on the tested feature; *—significant at *p* ≤ 0.05; **—highly significant *p* ≤ 0.01; ^n.s.^—not significant).

The share of available forms in the total content of the considered metals was strongly related to the experimental series (Figure 3).

In the case of Cd, these values ranged from 21.01% to 80.65%. The lowest share of the available forms of Cd was observed in series 4 (BA + Zn) and the highest was observed in series 2 (BA + Cd). The two remaining series were characterized by the share of available forms of Cd in the range of 21.35–21.78%. The BA doses used did not significantly alter the amount of Cd_av_ in Cd_tot_.

In turn, the average share of Pb_av_ content in Pb_tot_ in the individual experimental series ranged from 46.62% in series 2 (BA + Cd) to 71.40% in series 3 with lead acetate (BA + Pb). In series 1 (BA) and 2 (BA + Cd), increasing doses of BA did not significantly alter the indicated mean values. However, in series 3 (BA + Pb), the share of Pb_av_ in Pb_tot_ increased significantly at each BA dose. In series 4 (BA + Zn), the share of Pb_av_ in Pb_tot_ was significantly lower only after the first dose of BA (30 mg pot^−1^). The remaining BA doses had no significant effect on this feature.

In the case of Zn, the results obtained were different. The highest share of Zn_av_ in Zn_tot_ was observed in series 1 (only with BA), which amounted to 55.27%. However, in series 2 (BA + Cd) and 3 (BA + Pb), this share was significantly lower and amounted to 50.72% and 47.19%, respectively. The lowest share of Zn_av_ in Zn_tot_ was recorded in the series with simulated Zn pollution (13.89%) and was significantly lower in relation to the remaining series. In series 1 (only with BA), the share of Zn_av_ in Zn_tot_ increased significantly with increasing doses of BA (*r* = 0.681 **), and the LSD test indicated a significant effect of the first dose of BA (30 mg pot^−1^). However, in the series with the addition of Cd and Zn, the share of Zn_av_ in Zn_tot_ decreased significantly against the background of increasing doses of BA (−0.781 ** < *r* < −0.743 **).

## 4. Discussion

The BA used in the presented investigation was characterized by an alkaline reaction and a favorable sorption complex, as well as a relatively low salinity. Moreover, it contained significant amounts of C_tot_, K, Ca, Fe, Mn, and Zn. The mentioned properties contributed to the attempt to evaluate this waste in terms of its natural use as a soil improver. Currently, special attention is paid to the fertilizing potential of BA due to its abundance of nutrients such as Ca, K, and a number of microelements [3,4]. Currently, some researchers have determined the target dose of this material for cultivation purposes based on the K content in BA [14].

However, as the literature data indicate [2,4] the composition of BA can be very diverse, not only in terms of the content of macro- and micronutrients, such as Zn, but also in terms of the content of toxic elements, including: Cd and Pb. According to Szostek et al. [30], the composition of ashes after combustion of a mixture of wood and agricultural biomass, among those considered in the presented work, is dominated by Zn (470 mg kg^−1^). A much lower content level is found for Pb (37.63 mg kg^−1^), and the lowest is found for Cd (3.69 mg kg^−1^). Similar changes in the contents of these metals were also observed by Pazalja et al. [1] in pellet ashes. Importantly, according to Szostek et al. [30], these metals occur in the smallest amounts in the exchangeable fraction, which is the most mobile and the most available to plants. In the cases of Pb and Zn, the residual fraction has the largest share of their total contents, and, in the case of Cd, the reducible fraction.

Looking a bit more widely, we can assume that a high pH value and a sorption complex rich in base cations will enable the use of BA as a sorbent of heavy metals in soil with an increased content of heavy metals. This was the assumption behind our research, in which we observed a significant increase in the contents of Cd_tot_ and Cd_av_ in the soil and a significant decrease in the content of Zn_tot_ as a result of BA application (Table 1, Table 3 and Table 4). Importantly, this significant increase in the contents of Cd_tot_ and Cd_av_ correlated with BA doses (Table 1 and Table 4) did not correspond to an increase in the proportion of Cd_av_ in Cd_tot_ (Figure 3). In turn, the share of Pb_av_ in Pb_tot_ occurred after the introduction of the highest BA dose (90 g pot^−1^), and the share of Zn_av_ in Zn_tot_ decreased significantly at the BA doses of 60 and 90 g pot^−1^. The obtained results can be explained by the fact that BA application increased the soil reaction [4,8], and this results in the transformation of heavy metals, including Cd, Pb [31,32], and Zn [33] into unavailable (insoluble) forms. Moreover, the addition of BA increases soil porosity and aeration and thus improves the ability to conduct cation exchange and retain nutrients and water in soil [4]. BA application increases the C_tot_ content in the soil and increases CEC [8], which also promotes the immobilization of heavy metals in the soil [32,33]. Amendments that immobilize pollutants in the soil reduce the leaching of trace elements and, at the same time, their bioavailability [33,34]. This happens by inducing various sorption processes, including adsorption on mineral surfaces, surface precipitation, ion exchange, and the formation of stable complexes with organic ligands [33]. The retention of heavy metals by organic matter depends on many factors, among which soil pH plays an important role [33,34]. This indicator can be regulated by the use of BA [4,8]. The immobilization of Pb and Zn in soil is closely correlated with pH. The lowest mobility of Pb and Zn is observed under neutral to slightly alkaline reaction conditions [33]. According to Hamid et al. [13] alkaline additives increase the soil pH and the negative charge of the surface, thereby reducing the activity of heavy metals. As a result of these changes, obtained after the use of BA, an increase in the content of nutrients in the soil is observed [13], including available forms of P, K, and Mg [8,14].

When applying BA to the soil, attention should be paid to the balance of heavy metals in the soil, because the cultivation of edible plants in soil contaminated with toxic elements is not allowed [31]. The contamination of soil, especially agricultural soils, with heavy metals poses a risk not only to the environment but also to human health [11,13,35,36]. Soils contaminated with heavy metals should first be subjected to remediation processes, of which BA is a promising material [35]. A 3-year study by Szostek et al. [30] showed that the use of BA as a fertilizer significantly increased the Zn content in the soil but did not increase the concentrations of Cd and Pb. In turn, in the research of Wierzbowska et al. [14], only a slight increase in the content of Pb total form was observed. However, in the research by Rolka et al. [8] an increase in the contents of total Pb and Cd, an increase in the available forms of Zn and Pb, and a decrease in the content of available forms of Cd were observed. The current presented studies clearly demonstrate a close relationship between the contents of available forms of metals and their total content in the soil (Table 4). These relations were closely related to simulated soil contamination with the tested metals.

The high share of available forms of Cd, Pb, and Zn and their total forms in the presented research (Figure 2) should also be inextricably linked to the type of extractant used, which in this study was 1 mol dm^−3^ HCl. HCl is a very strong extractant because it releases metals associated not only with the exchangeable fraction but also with the carbonates and the organic and oxide fractions [34]. HCl was chosen deliberately to indicate the share of metal forms in the soil that may be potentially available to plants, i.e., forms that can be included in the biological cycle [34].

Although there is little data in the literature on the immobilization role of BA, nevertheless, when comparing the composition of BA with the composition of biochar or fly ash from hard coal, one can assume the high effectiveness of BA in this aspect. Hard coal fly ash like BA is characterized by an alkaline reaction, is rich in nutrients, and has a higher water retention capacity. The application of fly ash to soil usually improves the physical, chemical, and biological properties of soils [37,38,39]. Like the use of BA, the application of fly ash may also result in the release of trace elements into the environment or their immobilization. In the research by Ram and Masto [40], the observed process of the immobilization of heavy metals as a result of the use of fly ash was intensified by the addition of other mineral or organic substances. A good result in terms of Pb immobilization in the soil was caused by the use of fly ash together with peat, which, according to Kumpiene et al. [41] results in a significant reduction in the bioavailability of this metal. Importantly, such treatments can be used in situ to remediate Pb-contaminated soils [41].

In recent years, the available literature has paid more and more attention to the immobilization roles of a mixture of fly ash and biochar [42], biochar itself [43,44,45,46,47], and biochar with various minerals [36] or charcoal [48,49]. The mixture of coal ash and biochar showed an effective immobilization of Pb and Cd, which was mainly related to the increased pH value [42]. Biochar obtained from agricultural waste or plant residues produced in the pyrolysis process can act as an effective surface sorbent for Zn [44,47,50], Pb [46,47], and Cd, indicating the permanent retention of these metals [47,50]. Biochar significantly increases soil pH [10,35,43,47,51], increases CEC [43,51], and reduces the mobility of Cd and Zn [12,35]. However, the use of biochar may also have negative environmental effects, including the penetration of pollutants from biochar into the soil. Moreover, the economic benefits of using biochar for recultivation purposes are still not satisfactory [51]. A composite of biochar with minerals, which has a large specific surface area, porous structures, and various surface functional groups, has turned out to be effective in immobilizing metals [36]. According to Cui et al. [48], the addition of charcoal increases the contents of organic matter and total forms of Cd and reduces the amount of its CaCl_2_-extractable. However, the authors draw attention to the fact that when immobilizing heavy metals, it is very important to ensure the stability of soil aggregates, especially when the reclaimed area is used for agriculture. Interesting results can also be observed in the work of Ðurić et al. [52], in which the effective immobilization of Cd, Pb, and Zn in the soil was noted after the use of paper-ash. The immobilization of these metals occurred through the precipitation of insoluble hydroxides and carbonates. Creating permanent bonds of metals with paper-ash ingredients protects the environment against the possibility of further migration of these elements. Ashes from sewage sludge combustion activated with oxalic acid play a significant role as a material for immobilizing Pb in the soil [53], which can effectively reduce the leaching of Pb from the soil, precipitating it in the stable form of lead phosphate minerals. However, in the case of an excess of oxalic acid, phosphates and Zn may be washed out.

Similarly, in order to improve the immobilization properties of BA, combining this material with other byproducts that have stable compositions and are free from undesirable impurities can be considered. Such a mixture could include materials that will be additional sources of nitrogen, of which BA contains negligible amounts. There are also studies in which potential sorption materials are subjected to various modifications in order to improve their properties and thus improve immobilization effects. An example here is the research of Xu et al. [32], who subjected fly ash to various modifications in order to improve the immobilization effects concerning Pb and Cd. They achieved positive results after subjecting the ashes to low temperatures and hydrothermal treatments. Also, in the case of BA, an aspect worth considering seems to be the selection of the biomass combustion temperature, which may contribute to better quality in terms of heavy metal immobilization in the soil.

## 5. Conclusions

The application of the adopted BA doses to the soil with natural Cd, Pb, and Zn content (series 1) resulted in an increase in the Cd_tot_ and Cd_av_ contents and a decrease in Zn_tot_. However, a statistically significant increase was recorded for the content of Cd_tot_ when applying 90 g of BA pot^−1^. Despite these observations, the share of available forms of Cd and Pb in their total content did not change with respect to different doses of BA. The exception was the content of Zn_av_, the share of which in the total content increased as a result of the BA application. Simulated soil pollution with heavy metals resulted in a multiple increase in their total and available content, which was observed in all objects. As a result of these changes, an additional increase in the content of the available forms of Pb_av_ and a decrease in the content of Zn_av_ after BA application were observed. However, no significant increase in Cd_av_ content in the soil was detected. Similarly, the share of the total contents of the available forms of the above-mentioned metals was much higher in the soil with simulated pollution with these elements. However, in the context of increasing BA doses, no increase in the share of Cd_av_ in Cd_tot_ was observed. In the case of Pb, the increase in the share of Pb_av_ in Pb_tot_ occurred after the introduction of the highest BA dose (90 g pot^−1^), and the share of Zn_av_ in Zn_tot_ decreased at the BA doses of 60 and 90 g pot^−1^.

The statistical analysis of the results proves a strong correlation between the total content of the metals in question and their available forms. This fact was significantly influenced by the simulated soil contamination.

The obtained results indicate the immobilization effect of BA only with respect to zinc. The analyzed ashes were used on soils with increased metal content. However, the promising trends observed in the formation of Zn_av_ in the Zn_tot_ pool should be compared in the future with the possibility of using BA in attempts at the remediation of soils with higher levels of contamination and also in relation to other heavy metals.

## Figures and Tables

**Table 1:** Cd content in the soil (mg kg^−1^)—total forms.

BA Doses g pot^−1^	BA	BA + Cd	BA + Pb	BA + Zn	Mean for Doses
0	0.520 a	1.873 e	0.593 abc	0.627 abcd	0.903 a
	±0.028	±0.084	±0.041	±0.038	±0.019
30	0.533 a	1.840 e	0.600 abc	0.767 d	0.935 ab
	±0.038	±0.114	±0.033	±0.062	±0.058
60	0.567 ab	2.007 f	0.760 d	0.727 cd	1.015 c
	±0.025	±0.066	±0.033	±0.009	±0.023
90	0.733 cd	1.920 ef	0.627 abcd	0.693 bcd	0.993 bc
	±0.019	±0.150	±0.034	±0.034	±0.046
Mean for series	0.588 a	1.910 c	0.645 ab	0.703 b	0.962
	±0.090	±0.125	±0.076	±0.065	±0.060
*r*	0.887 **	0.274 ^n.s.^	0.381 ^n.s.^	0.274 ^n.s.^	0.070 ^n.s.^

As part of the LSD*_p_*_ ≤ 0.05_ analysis, different letters (a, b, c, d, ab, etc.) next to the given values indicate the significant impact of individual BA doses on the tested feature; **—highly significant *p* ≤ 0.01; ^n.s.^—not significant.

**Table 2:** Pb content in soil (mg kg^−1^)—total forms.

BA Doses g pot^−1^	BA	BA + Cd	BA + Pb	BA + Zn	Mean for Doses
0	12.49 a	13.50 ab	76.41 d	13.69 ab	29.02 b
	±0.58	±0.53	±1.83	±0.37	±0.55
30	12.58 a	13.05 ab	94.33 f	14.87 b	33.71 d
	±0.38	±0.21	±1.66	±0.31	±0.53
60	13.31 ab	13.51 ab	85.58 e	14.07 ab	31.62 c
	±0.14	±0.35	±1.15	±0.36	±0.25
90	13.12 ab	13.11 ab	70.99 c	14.44 ab	27.92 a
	±0.12	±0.04	±1.42	±0.00	±0.37
Mean for series	12.88 a	13.29 a	81.83 c	14.27 b	30.57
	±0.50	±0.40	±9.04	±0.53	±2.30
*r*	0.585 ^n.s.^	−0.200 ^n.s.^	−0.310 ^n.s.^	0.308 ^n.s.^	−0.020 ^n.s.^

As part of the LSD*_p_*_ ≤ 0.05_ analysis, different letters (a, b, c, d, e, f, ab) next to the given values indicate the significant impact of individual BA doses on the tested feature; ^n.s.^—not significant.

**Table 3:** Zn content in the soil (mg kg^−1^)—total forms.

BA Doses g pot^−1^	BA	BA + Cd	BA + Pb	BA + Zn	Mean for Doses
0	21.69 b	15.31 a	17.31 ab	188.0 c	60.59 a
	±0.90	±0.09	±0.50	±4.66	±1.31
30	18.96 ab	18.52 ab	17.64 ab	189.6 c	61.17 a
	±0.65	±0.16	±0.26	±5.46	±1.50
60	18.64 ab	19.06 ab	18.74 ab	189.8 c	61.57 a
	±0.47	±0.75	±0.41	±5.98	±1.29
90	18.69 ab	20.79 ab	20.90 ab	212.66 d	68.26 b
	±0.53	±0.44	±0.20	±1.68	±0.42
Mean for series	19.50 a	18.42 a	18.65 a	195.0 b	62.90
	±1.44	±2.03	±1.45	±11.26	±3.34
*r*	−0.728 *	0.935 **	0.915 **	0.737 *	0.034 ^n.s.^

As part of the LSD*_p_*_ ≤ 0.05_ analysis, different letters (a, b, c, d, ab) next to the given values indicate the significant impact of individual BA doses on the tested feature; *—significant at *p* ≤ 0.05; **—highly significant *p* ≤ 0.01; ^n.s.^—not significant.

**Table 4:** Cd content in soil (mg kg^−1^)—available forms.

BA Doses g pot^−1^	BA	BA + Cd	BA + Pb	BA + Zn	Mean for Doses
0	0.113 a	1.519 b	0.128 a	0.142 a	0.476 a
	±0.006	±0.044	±0.016	±0.016	±0.015
30	0.125 a	1.497 b	0.128 a	0.138 a	0.472 a
	±0.008	±0.069	±0.004	±0.001	±0.019
60	0.131 a	1.581 b	0.141 a	0.146 a	0.500 a
	±0.009	±0.140	±0.002	±0.003	±0.037
90	0.137 a	1.556 b	0.148 a	0.159 a	0.500 a
	±0.001	±0.094	±0.004	±0.001	±0.024
Mean for series	0.126 a	1.538 b	0.136 a	0.146 a	0.487
	±0.011	±0.099	±0.012	±0.012	±0.028
*r*	0.784 **	0.220 ^n.s.^	0.663 *	0.575 ^n.s.^	0.019 ^n.s.^

As part of the LSD*_p_*_ ≤ 0.05_ analysis, different letters (a, b) next to the given values indicate the significant impact of individual BA doses on the tested feature; *—significant at *p* ≤ 0.05; **—highly significant *p* ≤ 0.01; ^n.s.^—not significant.

**Table 5:** Pb content in the soil (mg kg^−1^)—available forms.

BA Doses g pot^−1^	BA	BA + Cd	BA + Pb	BA + Zn	Mean for Doses
0	6.031 a	5.905 a	46.69 b	7.174 a	16.45 b
	±0.143	±0.063	±3.03	±0.415	±0.71
30	6.430 a	6.218 a	63.53 d	6.619 a	20.70 a
	±0.063	±0.149	±1.56	±0.073	±0.36
60	6.536 a	6.224 a	61.85 c	6.806 a	20.35 a
	±0.374	±0.072	±2.76	±0.080	±0.76
90	6.432 a	6.412 a	60.15 c	6.994 a	20.00 a
	±0.146	±0.209	±3.66	±0.075	±0.89
Mean for series	6.357 a	6.190 a	58.06 b	6.899 a	19.38
	±0.289	±0.228	±7.26	±0.300	±1.85
*r*	0.507 ^n.s.^	0.730 **	0.596 ^n.s.^	−0.131 ^n.s.^	0.051 ^n.s.^

As part of the LSD*_p_*_ ≤ 0.05_ analysis, different letters (a, b, c, d) next to the given values indicate the significant impact of individual BA doses on the tested feature; **—highly significant *p* ≤ 0.01; ^n.s.^—not significant.

**Table 6:** Zn content in the soil (mg kg^−1^)—available forms.

BA Doses g pot^−1^	BA	BA + Cd	BA + Pb	BA + Zn	Mean for Doses
0	9.917 bcd	9.124 ab	8.167 a	28.68 f	13.97 a
	±0.49	±0.43	±0.73	±0.69	±0.47
30	11.00 d	9.549 abc	9.493 abc	26.35 e	14.10 a
	±0.46	±0.38	±0.55	±1.00	±0.16
60	11.08 d	8.808 ab	8.569 ab	26.68 e	13.79 a
	±0.25	±0.96	±0.24	±1.29	±0.15
90	10.79 cd	9.412 abc	8.806 ab	26.09 e	13.78 a
	±0.60	±0.60	±0.15	±0.38	±0.17
Mean for series	10.70 b	9.223 a	8.759 a	26.95 c	13.91
	±0.66	±0.69	±0.68	±1.36	±0.30
*r*	0.460 ^n.s.^	0.020 ^n.s.^	0.164 ^n.s.^	−0.609 ^n.s.^	−0.013 ^n.s.^

As part of the LSD*_p_*_ ≤ 0.05_ analysis, different letters (a, b, c, d, abc, etc.) next to the given values indicate the significant impact of individual BA doses on the tested feature; ^n.s.^—not significant.

**Table 7:** Correlation between the contents of the total and available forms of heavy metals.

Elements	Cd_tot_	Cd_av_	Pb_tot_	Pb_av_	Zn_tot_
Cd_av_	0.988 **				
Pb_tot_	−0.325 **	−0.332 **			
Pb_av_	−0.328 **	−0.335 **	0.986 **		
Zn_tot_	−0.271 ^n.s.^	−0.325 **	−0.316 **	−0.318 **	
Zn_av_	−0.295 **	−0.342 **	−0.368 **	−0.371 **	0.982 **

**—highly significant *p* ≤ 0.01; ^n.s.^—not significant.

## Data Availability

Data are contained within the article.
